# Cutaneous Discolorations

**Published:** 1861-03

**Authors:** 


### CUTANEOUS DISCOLORATIONS.

Dr. Laycock, in the *British and Foreign Review*, gives the following conclusions:

1. That besides blue and green, of rare occurrence, there are two common well-marked and distinct forms of morbid discoloration due to pigment deposit—the *yellow* or sallow, and the black or *swarthy*.

2. That both yellow and swarthy discoloration of the skin will occur from the action of local irritants—as heat, light, cutaneous parasitic fungi, blisters, sinapisms, and the like, or in the progress of various cutaneous diseases of the skin and its appendages.

3. That the absence of pigment (leucopathia), as well as its deposits, may be caused by inflammatory and other diseases of the skin, affecting its chromatogenous function.

4. That morbid states of the cerebro-spinal centres will influence the deposit or non-deposit of pigment.

5. That morbid states of the genito-urinary organs in both sexes, acting probably through the nervous system, will determine the election of locality of pigment-deposit, according to the same law by which the development of sexual hair and pigment is regulated.

6. That structural diseases of the abdominal viscera and peritoneum also exercise an influence through the nervous system upon the local deposit of pigment in the skin.

7. That in disease of the supra-renal capsules, the bronzing of the skin, whether swarthy or yellow, is partly nervous, and due to the direct or indirect influence of the capsules or the kidneys and nervous system; partly hæmic, and in so far due to the morbid influence of "dyscrasic" blood.

8. That pigmentary changes in the skin of both whites and blacks may be *result* of morbid causes, and yet may remain after the operation of the causes has ceased, and assume a physiological character.

9. That although local morbid pigmentation of the skin may occur exclusively from local causes, or the influence of the nervous system, in the majority of cases there is a morbid condition of the blood.

10. That the morbid conditions of the blood associated most commonly with pigmentary changes are characterized by those changes in the blood-corpuscles (leukæmia, leucocytosis)

which are observed in cachectic states of a constitutional character (pregnancy, chlorosis, tertiary syphilis, chronic rheumatism, cancer, etc.), or which are intimately connected with "dyscrasic," visceral, or glandular diseases (of the spleen, suprarenal capsules, lymphatic glands).

11. That the tendency to discoloration increases (*cæteris paribus*) with age after a certain period of life.

12. That the morbid pigment-deposits proper, as distinguished from masses of altered blood-corpuscles, are carbonaceous excretions, and are often vicarious with the suspension or imperfect elimination of other carbonaceous excretions—as the carbonic and lactic acids, and the pigment constituents of both the urine and bile; and are consequently associated with morbid states of assimilation, as well as of elimination (through the skin, lungs, liver, kidneys).

13. That amongst the morbid states of assimilation, the rheumatic and gouty are specially to be classed, as well as those coincident with anæmia.

*Semeiology.*—Pigmentary changes in the skin, and pigment-deposits in the tissues, are observed clinically under the most varied conditions, and have given trivial names to groups of symptoms. *Jaundice* (*jaune*, yellow,) is the simplest illustration of these. The deposit of black, or brown, or blue pigment in the skin of white races has led to the use of various nosological terms indicative of the change—as *melasma*, *melanopathia*, *nigrities*, *bronzed skin*, blue skin, or *cyanopathia*, *meliceris*, *stearrhœa*, *flavescens*, *stearrhœa nigricans*, chlorosis (or *green* sickness,) *melancholia*, *melanchlorosis*, *melasicterus*, etc. As to the absence of pigment, we have *albura*, *leuce*, *leucopathia*, *vitiligo*, *canities*, etc. The congenital absence known as albinism has always excited curious attention, and, as those who have treated albinos know, coincides with peculiar forms of disease. I need not refer to the albino forms of animals, nor to the curious ethnological doctrines and oppressive laws which have originated in the presence or absence of cutaneous pigment, except to say that a better knowledge of the pathological forms will necessarily throw much light on the physiological.

*Classification of Morbid Pigments.*—The pathological pigments are of two kinds. 1. The *spurious*, which consists either in foreign carbonaceous matters, or in direct modifications of the coloring matter of the blood-corpuscles after they have died; these pigments are all some form of hæmatine, and present all the shades of black, brown, yellow, and purple.

2. The *true*, being those pigments which are products of the transformation of the living blood-corpuscles, or tissues, and which must be held to differ from the preceding in the circumstance that they are the results of the action of the vital forces. They are of all colors; correspond in this respect to the normal coloring matters of animals; and are found in the cutaneous appendages and excreta, but especially in the urine and bile.

Le Cat, a surgeon at Rouen, was the first to examine systematically the morbid pigmentary changes of the human skin in their relation to anatomy, physiology, and organic chemistry. He details cases of melasma and nigrities, and distinguished what was evidently a case of "bronzed skin" from ordinary melasma and icterus.* He examined the pigment (which he termed "Æthiop's mineral") chemically, and showed that the coloring matter of the ink of the cuttle fish was identical in nature with that of the skin of negroes, and of the choroid coat of the eye. He was also the first to observe that the encephalic tissues of the negro were of a darker tint than in whites—an observation subsequently confirmed by Meckel and others, and very recently by M. Gubler. Although considerable progress in observation has been made during the last century, we may still say, with Alibert, "Les lois de la coloration sont encore couvertes d'un voile epais."

Modern inquiries have ascertained that black pigment is deposited morbidly in the tissues, mucous membranes, and capillaries (melanosis), as well as in the skin (melasma), and that it is sometimes present in considerable quantity in the blood (melanæmia). Its nature and composition have also been carefully examined of late years. Barruel first attempted to show that the chemical composition of the black deposit in melanosis was identical with that of the colouring matter of the blood. Breschet founded upon this analysis and upon his own researches the conclusion that the deposit was due to effused and modified blood with a large proportion of true colouring matter; and Heusinger, Lobstein, Andral, Trousseau, and Leblane, J. Vogel, Bruch, Rotkitansky, Virchow, and others, have theorised as to the mode in which the pigment is formed from the blood.† It is now well established

* Traité de la Couleur de la Peau Humaine, &c., p. 158. 1765.

† Compare Rokitansky's Pathol. Anatomy, Sydenham Society's translation, vol. i. p. 204, and Virchow's elaborate paper. Die Pathol. Pigmente, in Archiv f. Path. Anatomie und Physiologie, vol. i. art. 9.

that although the pigment in numerous cases really consists of modified hæmatine, derived directly from the blood-corpuscles, the deposit in melanosis, melasma, and nigrities is not of this kind.

